# Welcome Message from the Scientific Programme Committee

**DOI:** 10.2196/jmir.1.suppl1.e1

**Published:** 1999-09-19

**Authors:** Theorodos N Arvanitis, Gunther Eysenbach, Jack Woodall

## Abstract

It gives us great pleasure to welcome you to MEDNET99 - The 4th World Congress on the Internet in Medicine.

MEDNET99 is an international meeting which aims to bring together researchers, developers and users involved in the application of the Internet in Medicine. The Congress - this year under the theme "Towards the Millennium of Cybermedicine - will provide a forum for exploration of the rapidly developing relationship between medical sciences and the Internet, and is relevant to all medical and health care professionals, as well as those involved in the development and application of the new technological opportunities offered to the medical field by the Internet and the World Wide Web.

This year's Congress will take place over three full days, 19th - 21st September 1999, in the small and beautiful town of Heidelberg, one of the most famous tourist destinations in Germany and the town with the oldest German University.

MEDNET99 follows on from three successful MEDNET meetings, all held in the United Kingdom, which were attended by large international and multi-disciplinary groups of delegates. MEDNET98 was attended by more than 250 participants. After these three successful conferences in the United Kingdom, this year's MEDNET will for the first time be hosted outside of the UK. It will therefore hopefully be the first in a long series of World Congresses of Internet in Medicine hosted in different countries around the globe.

MEDNET99 will be preceded by several events on Saturday, 18th September: In a series of tutorials, experts in the field share their knowledge. To take account of local developments in the Internet scene we also organised a satellite symposium on "Internet and Medicine in Germany". A further novum is the "patient afternoon", where we bring together health care professionals with consumers to talk about medical information — in particular cancer information — on the Internet.

MEDNET99 will be focusing on the following themes:

- applications of the Internet in medical information exchange and medical education
- role of Internet and information exchange in evidence-based healthcare
- use of the Internet for consumer health promotion and impact of the Internet on public health
- global and regional health information networks
- commercial information systems and portal sites for physicians
- eJournals and scientific publishing on the Internet, online peer-review, impact of Internet on traditional medical publishing
- telemedicine
- search engines, search agents and other search tools
- electronic mail
- using the Internet for medical research and clinical trials
- quality of Internet information
- Internet usage statistics and demographics
- clinical information systems and Intranets
- system design issues
- legal and ethical issues

As well as providing a forum for delegates to exchange valuable information, MEDNET99 will provide a varied program of social events and interesting workshops.

On behalf of the organisers and the Scientific Programme Committee of MEDNET99, we would like to extend a warm welcome to Heidelberg and we hope that you will all have a stimulating and rewarding Congress.

